# Assessing zygosity in progeny of transgenic plants: current methods and perspectives

**DOI:** 10.14440/jbm.2016.114

**Published:** 2016-07-18

**Authors:** Nishat Passricha, Shabnam Saifi, Surender Khatodia, Narendra Tuteja

**Affiliations:** ^1^Plant Molecular Biology Group, International Centre for Genetic Engineering and Biotechnology, Aruna Asaf Ali Marg, New Delhi 110067, India; ^2^Amity Institute of Biotechnology, Amity University, Gurgaon 122413, India; ^2^Amity Institute of Microbial Technology, Amity University, Noida 201313, India

**Keywords:** digital PCR, homozygous line, LAMP assay, real-time PCR, transgenic plants

## Abstract

Homozygosity is highly desirable in transgenic plants research to ensure the stable integration and inheritance of transgene(s). Simple, reliable and high-throughput techniques to detect the zygosity of transgenic events in plants are invaluable tools for biotechnology and plant breeding companies. Currently, a number of basic techniques are being used to determine the zygosity of transgenic plants in T_1_ generation. For successful application of any technique, precision and simplicity of approach combined with the power of resolution are important parameters. On the basis of simplicity, resolution and cost involved, the available techniques have been classified into three major classes which are conventional methods, current methods and next generation methods. Conventional methods include antibiotic marker-based selection and the highly labor intensive Southern blot analysis. In contrast, methods such as real time PCR, TAIL PCR and competitive PCR are not only cost effective but rapid as well. Moreover, methods such as NGS, digital PCR and loop-mediated isothermal amplification also provide a cost effective, fast and not so labor intensive substitute of current methods. In this review, we have attempted to compare and contrast all the available efficient methods to distinguish homozygous plants in progeny of transgenics. This review also provides information of various techniques available for determining zygosity in plants so as to permit researchers to make informed choices of techniques that best suit their analyses. More importantly, detection and subsequent selection of homozygous individuals is central for facilitating the movement of transgenic plants from the laboratory to the field.

A diploid organism is homozygous at a gene locus when two identical alleles of the gene are present. A plant is called homozygous for a particular gene when both alleles at given locus are similar (dominant or recessive). A homozygous plant maintains a high degree of consistency for particular characters determined by the gene throughout the subsequent generations (true to type progenies-pure lines). In a breeding program, homozygous lines have many advantages such as uniformity in maturity, height, texture and canning qualities [1]. High yielding pure line (homozygous) method of breeding crop varieties is therefore preferred by the plant breeders wherever feasible. In recent era, transformation methods have hastened the plant research and gene characterization by using reverse genetic approach as a pivotal path for crop improvement. Transformation offers novel means to manipulate plant genome to withstand biotic and abiotic stresses, to produce secondary metabolites and other novel alkaloids which are useful as pharmaceuticals [2]. Therefore, in the last two decades, role of transgenics has increased in both basic and applied studies in plant biology. Production of transgenic plants has been reported including [3] tobacco [4], cotton [5], maize [6], rice [7], tomato [8] and *Arabidopsis thaliana* [9] etc.

With the advent of new technologies, several methods have also been developed for plant transformation. These methods have several variations and generally are crop specific. *A. thaliana*, a model plant that can be easily transformed with either vacuum infiltration [10] or floral dip [11] method. Similarly, several reports have been published for rice, like *Agrobacterium* callus regeneration method [12] *in-planta* infection [13, 14] protoplast transformation [15] particle bombardment [16] and gene-gun [17] etc. Worldwide researchers have produced several transgenic plants but only few of them are commercially applicable. Transformation technology is very successful in raising the transgenics but it may produce multiple gene insertions in parent genome. Establishing transgenic plants is still an expensive, time consuming and tedious job. It can take months to years, to produce a small fraction of the homozygous transgenic seeds that are useful in research. Whenever T_0_ transgenic plants undergo sexual reproduction “selfing”, they give rise to plants of diverse genotypic constitutions - hemizygous, homozygous and negative for the transgene (**Fig. 1**). As per Mendelian genetics, single gene segregating population (monohybrid) produce 1:2:1 (genotypic ratio) for transgene. This indicates that only 25% plants are homozygous for transgene (dominant or recessive forms each) and are further useful for crop improvement. There are few reports where heterozygous and homozygous plants may have different phenotypes (depending on degree of dominance) and or due to a transgene dosage effect [18]. Therefore, it is necessary to identify homozygous and heterozygous plants among the descendants of each original transgenic plant.

However, the inability to predict the integration site and copy number of transgene will remain a limitation of transformation technology. Genome editing systems like CRISPR/Cas9 [19], transcription activator-like effector nuclease, TALEN, [20] and Cre-lox [21] have overcome this limitation. T_0_ transgenic plants are usually heterozygous or hemizygous for the transgene. Identification of homozygous plant for transgene is mandatory for product development, to ensure regulatory compliance and to guarantee traceability. So it has become necessary for biotechnology companies and breeders to determine the zygosity of transgenic events. The regular transgene transmission as well as its expression is a main prerequisite for the production of new cultivars. Therefore, the knowledge of segregation distortion frequency and the sources of this phenomenon have a substantial importance for breeding of transgenic varieties. The present paper attempts to review various methods of homozygous line identification in transgenic plants.

**Figure 1.**
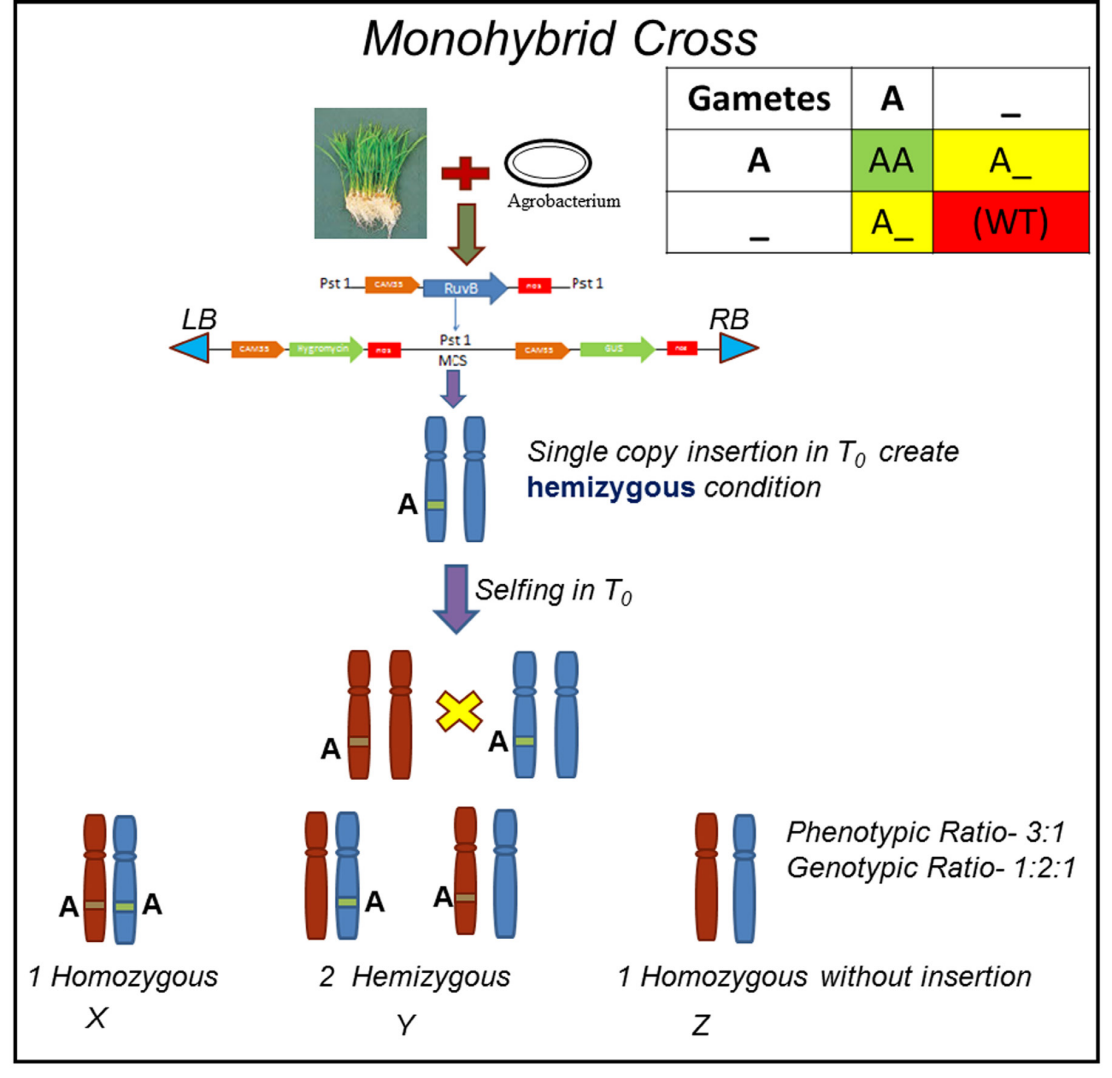
**Segregation of transgene in T_0_ generation and possible genotypes of transgene obtained in T_1_ population.** X, homozygous; Y, hemizygous and Z, wild type progeny.

**Figure 2.**
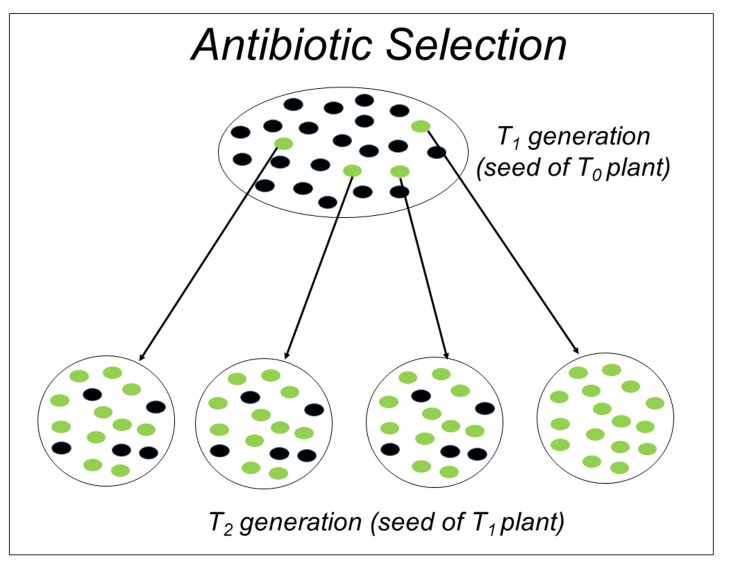
Schematic representation for antibiotic selection of transgenic plants, green color represents germinated seeds whereas black color represents non germinated seeds. Seeds of germinated positive plant (shown in green color) undergo for another round of selection on antibiotic medium. Setup of parent plant showing complete germination of seed indicates the homozygosity of transgene.

## 1. METHODS FOR ADVANCEMENT OF TRANSGENIC LINES

Genetic transformation provides a rapid method to alter the plant genome. In most of the techniques the desired gene is inserted randomly into few cells. These transformed cells are selected using particular marker and cultured to regenerate new transgenic plants. The first step after successive transformation is the confirmation of transgene insertion. To confirm the presence of transgene, polymerase chain reaction (PCR) and Southern blotting are the commonly used techniques but sometimes special phenotypic variation may also validate the transgene [22]. Effect of transgene is appeared through protein expression which can be verified through Western blotting and quantified by the levels of transgene expression by enzyme-linked immunosorbent assay (ELISA) etc.

Integration of foreign gene into a given locus is called a transgenic event. Transgenic (T_0_) plants generated (except microspore regeneration) immediately after the process of genetic transformation are hemizygous for the gene of interest. This transgene generally inherits a dominant trait [23,24], follows the Mendalian inheritance and produce homozygous, hemizygous plants after segregation [25-27]. As we are examining the homozygous plants 25% of the plants in the T_1_ population are useful for further work. To overcome the expenditure and save the time, plant biotechnology and breeding companies are seeking for simple and reliable, high-throughput techniques to detect the zygosity of transgene [28].

### Methods for zygosity testing

Resolution power is the ability of a technique to differentiate between the hemizygous and homozygous line. There are various methods available for testing the zygosity of transgenic plants. Based on resolution power and facilitation of a technique all the available methods have been categorized into three different classes.

#### Conventional methods

These methods have low resolution power to differentiate between hemizygous and homozygous line.

**(a) Antibiotic selection**: The basis of antibiotic selection depends upon two conditions, one the transgene should behave as dominant trait and second it should follow the Mendel first law of segregation. The seeds of T_0_ transgenic plants are selected on the plate of particular antibiotic marker (*e.g.*, hygromycin or kanamycin) present in vector used for transformation. The seeds collected from individual plants and plate few seeds of each plant on selection media (Murashige and Skoog Medium with antibiotic) (**Fig. 2**). Count the number of germinating seedlings in T_1_ population of each plant. As stated earlier antibiotic marker is dominant, it gives 75% of the progeny resistant for a single insertion. Anything above 0.75 will be considered as multiple insertion and rejected. The plate showing 100% germination indicates that resistant gene (transgene) is present in all the seed, implying that its parents must have a homozygous condition.

**(b) Southern blot analysis**: This is a routinely used technique to determine transgene copy numbers in the T_0_ generation [29]. By extension of experiment to T_1_ generation zygosity of plants can be also determined. By Southern analysis we can retrieve the information on zygosity as well as the fingerprint of the integration event [30].

**Figure 3.**
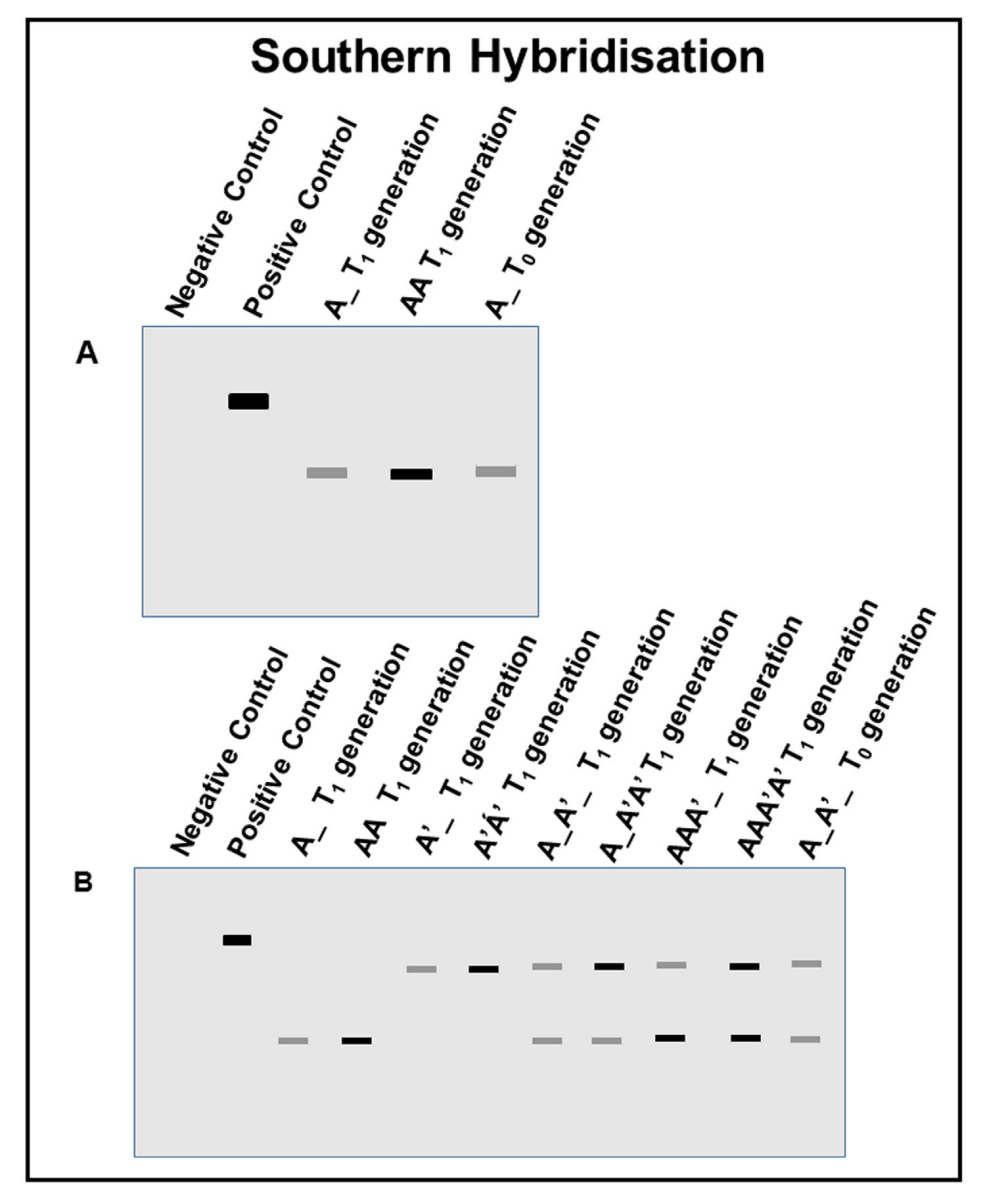
**Pictorial representation of Southern blot analysis showing difference in intensity of transgene blot for homozygous and hemizygous plant DNA. A.** Two possible combinations of monohybrid cross (single site insertion) showing homozygous and hemizygous conditions for transgene. **B.** All possible combinations for transgene integration at two different positions in plant genome at T_1_ generation.

For single insertional event: When gene A is inserted in the genome of a plant at a single position (locus), it follows the genotypic 1:2:1 segregation ratio in T_1_ generation (as explained in **Fig. 1**). Homozygous plant has two copies of gene in their genome so it hybridizes with more molecules of probes during the experiment and show brighter bands in the picture as compared to hemizygous line. This difference in intensity can be measured with ImageJ (https://imagej.nih.gov/ij/) software. This difference in value for band intensity indicates the zygosity of plant (**Fig. 3A**).

For multiple insertional event: When a single gene undergoes more than one (two in given cases) insertional event in the genome of same plant, it shows 4 different segregants in the phenotypic ratio of 9:3:3:1. In this case there are 8 possible combinations of gene arrangement as shown in **Figure 3B**. This shows the differences in the intensity between homozygous and hemizygous for one location of gene (**Fig. 3B**). Similarly, this difference in intensity can be noticed at other location of transgene.

#### Current methods

These methods have moderate range of resolution power.

**(a) Competitive PCR**: PCR is one of the most sensitive method for detecting the integrated gene in the transgenic plant genome. Thus, it can reduce the amount of template DNA required for analysis [31]. The copy number and the genotype determined by this method were identical to those estimated by Southern blot analysis and a segregation test (antibiotic selection).

**Figure 4.**
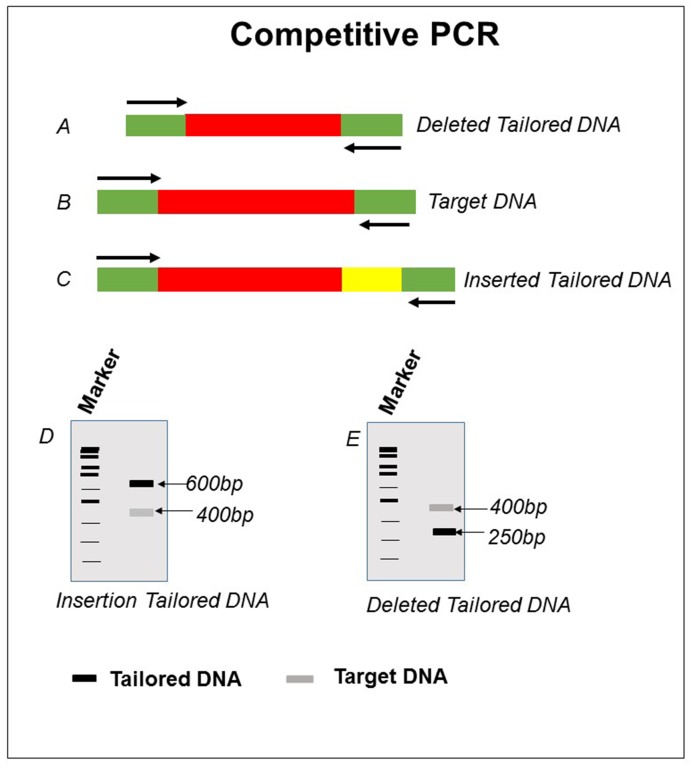
**Schematic representation of competitive PCR for target DNA (B) with deleted and inserted tailored DNA (A and C).** Respective results obtained is shown in pictorial view of gel (D and E).

*Principle.* When the two different DNA (target and competitor) are amplified together by the same set of primers in the same reaction tube, both templates will compete for amplification. Because of this competition, the ratio of the amounts of the two amplified products reflects the ratio of the amounts of the target DNA and its competitor (**Fig. 4**).

In competitive PCR, a known concentration of a tailored DNA fragment (competitor) is added to the reaction mixture containing target plant DNA. To amplify the tailored DNA, use the same set of primers as target DNA. Since the initial amount of the competitor is known, the amount of the target DNA can then be estimated according to the T:C ratio (T: amount of amplified product from target DNA; C: amount of amplified product from competitor). When the T:C ratio = 1, the initial amount of target DNA will correspond to the amount of competitor.

*Procedure.* Use the amplicon (**Fig. 4D** and **4E**) of tailored DNA (either deletion or insertion shown in **Fig. 4A** and **4C**, respectively) and the amplicon of target DNA for quantification using ImageJ (https://imagej.nih.gov/ij/) software. First quantitate the amplicon of target DNA of T_0_ generation with respect to tailored DNA amplicon. Use the same tailored DNA for quantification of target DNA of T_1_ generation (same plant).

*Results*. A plant is considered homozygous if target DNA (T_1_ generation) amplicon contains the two times the value of intensity as compared to T_0_ generation.

*Precaution*. Tailored and target DNA should be amplified by the same set of primers. Amplicon size of designed DNA should be distinguishable from the target DNA (different size, different restriction fragment pattern, etc.).

**(b) Real-time PCR**: Apart from testing of transgenics [32] quantitative real-time PCR (qPCR) has also been applied to reckoning of transgene. In addition, it can be a pragmatic tool for estimating the zygosity condition of transgene [33, 34]. Real-time PCR detects signal of reporters (SYBR Green or TaqMan) during PCR products accumulation. During the early cycles of amplification, the fluorescence level is low, but at a critical point fluorescence accumulates to a significant level perceived by the instrument’s detection system. This point, called the threshold cycle (C_t_), depends primarily on the starting amounts of nucleic acid [35]. The higher the initial amount of nucleic acid in the reaction, the smaller the C_t_.

*Method*.

ΔCT (tra) = Ct_tra_ – Ct_ref_

ΔCT (WT/T_0_) = Ct_tra_ – Ct_ref_

ΔΔCt= ΔCT (Tra) – ΔCT (WT/T_0_)

Copy number of the transgene by using formula = 2^–ΔΔCT^

[Ct_ref_ threshold cycles of reference gene; Ct_tra_: threshold cycles of the transgene; Ct_WT_: threshold cycles of the wild type; ΔCT: Difference between C_t_ value of candidate gene and reference gene; ΔΔCt: difference of ΔCT value of transgenic (sample) and wild type (control) plant

The above method can be used to determine the copy number of a transgene in T_0_ plant. To determine the copy number in T_0_ plants, use wild type as negative control and single copy endogenous gene as a reference *e.g.* sucrose phosphate synthase in rice [36], *Epsilon Cyclase* in wheat [34] and *Invertase* gene in tomato [18,37]. The multiple fold directly reflects the number of copies. To determine the homozygosity in T_1_ plant, use DNA of T_0_ plant as a control sample and choose any internal gene (*Actin, Tubulin* and *Ubiquitin*) as a reference gene. In this case, the reference gene is used to normalize the value obtained for transgene. Plant showing exactly double the copy number as compared to parent T_0_ plant, is considered as homozygous in nature.

Real-time PCR has been successfully employed to determine the zygosity/copy numbers in plants such as wheat [38,39], maize [40], rice [41], tomato [33] and sugarcane [42].

**Figure 5.**
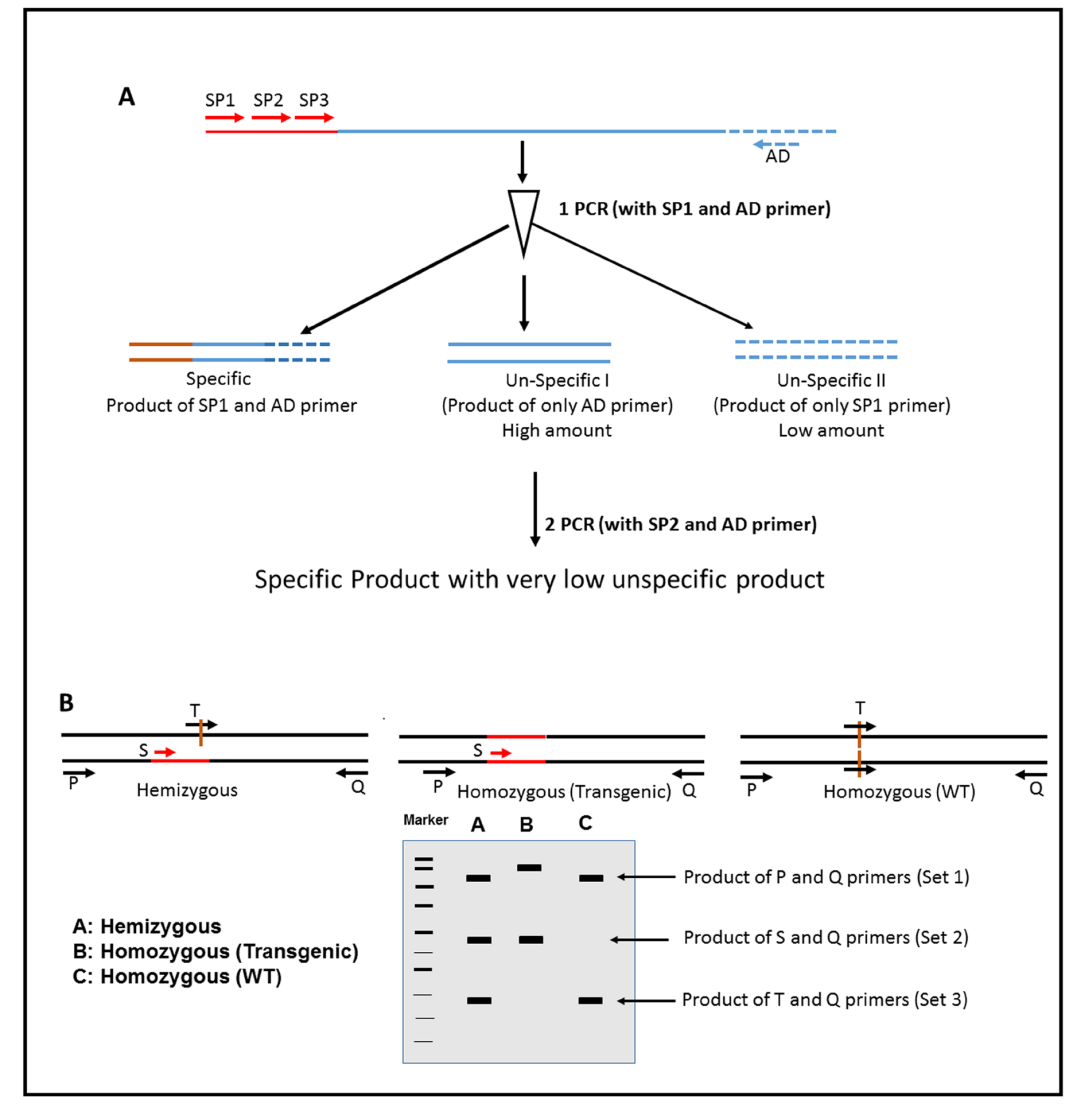
**Schematic representation of TAIL PCR for homozygosity detection. A.** Different PCR steps involved in TAIL PCR to identify the 3’ and 5’ sequence near the transgene (red colour). **B.** Line diagram of gel picture showing no of amplicon and their size for different zygosity status of plants.

**(c) TAIL PCR:** Thermal asymmetric intercalated (TAIL) PCR is an efficient technique to amplify the regions flanking a transgene [43-45]. This PCR strategy makes use of nested transgene-specific primers together with an arbitrary degenerate (AD) primer to amplify the unknown genomic DNA region flanking the insertion site. Priming by the AD and gene-specific primers create both specific (from the genic region) and unknown (from the region flanking the transgene insertion site) products. To control the amplification of the unknown PCR products, the PCR reactions need to be thermally optimized. In TAIL PCR, three serial PCR reactions have to be performed with nested primers for minimizing the amplification of unknown products (**Fig. 5A**). During the next reaction, PCR products from first PCR reaction are gradually diluted so that after final PCR reaction only specific products are detectable on the gel. Since TAIL-PCR is an extremely valuable and versatile tool for amplifying the sequences flanking a transgene insertion site and the subsequent identification of 3’ and 5’ regions flanking transgene insertion site. This information can then be used for evaluating the zygosity status in transgenic plants.

*Primer design*. Primers P and Q will be designed from identified flanking regions (5’ and 3’) by TAIL-PCR, respectively. Primer S is transgene specific, and primer T will be designed from the junction of the transgene (**Fig. 5B**).

*Method.* To detect zygosity status of transgenic plants, PCR amplification will be done with above mentioned 4 primers in a single PCR reaction. This PCR can generate a maximum of three amplicons that differ sufficiently in size to be distinguished by agarose gel electrophoresis [46] (**Fig. 5B**).

*Data analysis*.

(1) Hemizygous type: All the 4 primers will work in this condition. Primer set 1 (PQ) will anneal both to upstream and downstream region of the transgene insertion site to produce the largest amplicon. Primer set 2 (SQ) will work since there is presence of one copy of transgene in genomic DNA and it will give an amplicon of medium size. At the same time primer set 3 (TQ) are capable of amplifying the non-disrupted copy of genome and will produce the smallest amplicon. Presence of these three amplicons with different size identifies the hemizygous status of the transgene.

(2) Homozygous plant (transgenic): In this case both copies of genes are disrupted with insertion of the transgene at a specific site. So only two sets of primers PQ and SQ (primer set 1 and 2) will bind and are capable of amplifying a product.

(3) Homozygous (wild type): In this condition since the wild type gene is intact, hence only primer sets PQ and TQ (1 and 3) will anneal to generate the largest and smallest amplicon and thus confirming the homozygosity of transgene in plants.

**Figure 6.**
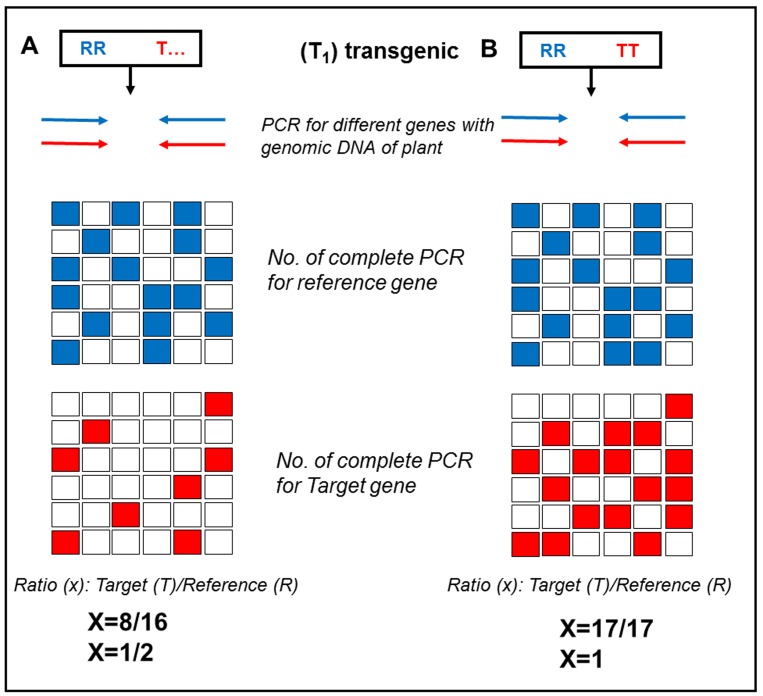
**Results obtained after digital PCR testing for homozygosity of transgene. A.** Result for hemizygous condition where ratio of positive reaction for reference gene (blue) to transgene (red) remain 0.5. **B.** Result for homozygous condition where ratio of positive reaction for reference gene (blue) to transgene (red) remain 1.

**Figure 7.**
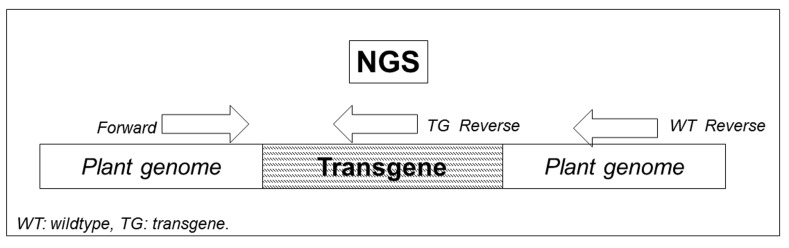
**Line diagram showing position of transgene in plant genome and the primer binding sites used to generate real time PCR and NGS templates.** The forward primer binds upstream of the transgene, one reverse primer binds within the transgene and another reverse primer showing binding downstream of the transgene.

**Figure 8.**
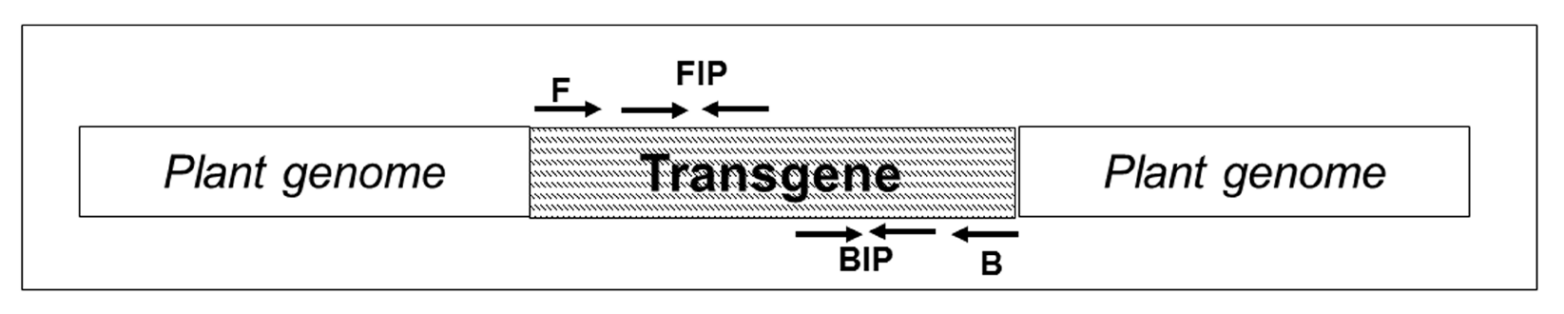
**Schematic representation of transgene insertion and position of primers used in LAMP assay.** F, forward outer primer; B, backward outer primer; FIP, forward inner primer; BIP, backward inner primer

**Figure 9.**
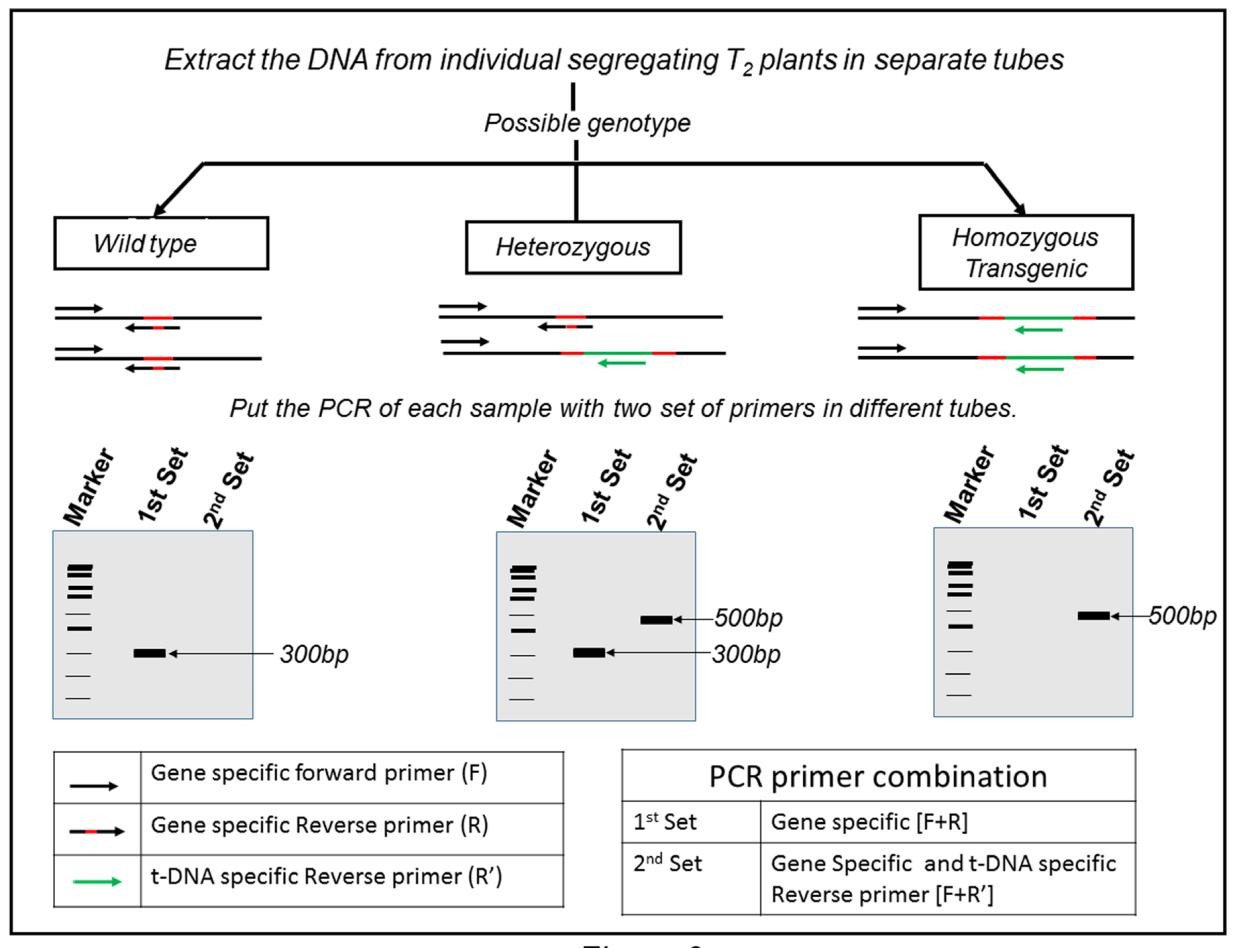
**Schematic representation of all possible combinations of insertional mutant in T_1_ generation.** Red color represents the site of insertion for T-DNA, green is symbolizing the inserted T-DNA sequence. If the target plant is wild type (no insertion mutation), a smaller PCR product (300 bp) appears on agarose gel. If the plant is heterozygous then PCR amplification shows two different sizes of band and for homozygous plant a single larger band (500 bp) appears.

**Figure 10.**
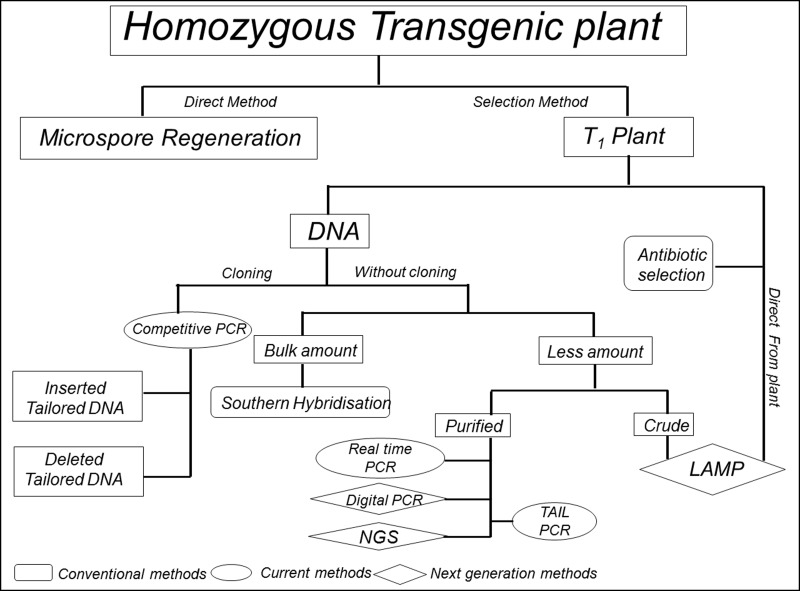
**A flowchart for various methods used to find the homozygous lines discussed in this review.** In this figure techniques have been classified based on their specific requirement for assay and advancement with time.

## 3. NEXT GENERATION TECHNIQUES

These methods have very high resolution power so their results are comparatively reliable than conventional and current methods.

### a) Digital PCR (dPCR)

In most of the available qPCR methods the quantification cycle (Cq) value depends on a number of factors, *e.g.*, instrument properties, reporter dye and assay efficiency, whereas dPCR depends on a simple count for the number of successful amplification reactions. Ideal dPCR does not require a calibration curve to determine the copy number, this can be calculated by counting of positive partitions in a reaction. Thus scientifically dPCR is generally more reproducible than other qPCR [43]. dPCR offers repeatability and reproducibility because it is less susceptible to inhibition and other external factor than qPCR.

*Procedure.* To find out the copy number of a transgene, take the DNA of target plant and digest with a zero cutter restriction enzyme of the transgene. After the digestion, add to the reaction primers of the target gene and reference gene (using optimized dyes FAM, ROX, and VIC dyes available from Life Technologies or Taqman) and interpret the results by comparing the ratio of desired gene with respect to reference gene. To determine the copy number (single or multiple insertions) of exogenous transgene, take a well-known gene as reference and amplify the specific loci (target and reference) by their respective primers. As shown in **Table 1** the ratio obtained will directly reflect the number of copies of transgene present in the genome of tested plant.

In case of an endogenous gene (gene of a plant is transformed back to same plant, also known as cis-genic) use the DNA of wild type plant to nullify the effect of the already present gene. In this case, first subtract the positive reactions obtained in the wild type plant from the transgenic plant and then calculate the ratio as in above case.

For finding the homozygous line, use the reference gene in the progeny population of a T_0_ plant. The possible condition of transgene in T_1_ population is [T_ and TT] hemizygous or homozygous (**Fig. 6A** and **6B** respectively) for the desired gene.

### b) Next-generation sequencing

Next-generation sequencing (NGS) is a robust approach to sequence entire genomes by putting together the large number of short sequence reads after assembling them into larger contigs. This technique helps strongly in high throughput detection of zygosity in transgenic plants and also in providing precise quantitative data for sequencing approaches based on census. In 2010, Metzker [44] used resequencing to calculate the frequency of specific alleles in a population and recently in 2013, Yockteng *et al* [45] used RNA-seq to determine accurate gene expression levels.

The zygosity determined by NGS at the transgene integration site is more accurate and reliable than those generated by PCR-based methods. NGS covered the 5’ border of the transgenic integration events or the genomic sequences at the wild-type locus (**Fig. 7**) and provided more reliable confirmation for the presence of each allele by covering more than 100 reads for a single allele. NGS could be adopted for high-throughput zygosity determination in transgenic plants by simultaneously processing a large number of samples. The only limitation of NGS assay is the requirement of the exact integration site and adjacent genomic sequences.

### c) Loop-mediated isothermal amplification (LAMP) assay

The LAMP assay is a rapid DNA amplification method called loop-mediated isothermal amplification [46]. The DNA polymerase used for LAMP has strand displacement activity. LAMP can amplify DNA under isothermal conditions ranging from 60 to 68°C within 1 hour by using a set of four specially designed primers. The amplified products can be visualized using gel electrophoresis, turbidity test and DNA-intercalating dyes like ethidium bromide and SYBR [47]. Positive amplicons from LAMP have been also observed with naked eye by adding metal indicators prior to the reaction such as magnesium sulphate (MgSO4), calcium chloride (CaCl_2_), propidium iodide, hydroxynaphthol blue (HNB), phenol red [48].

*Procedure.* Initially, DNA is added to LAMP mixture with forward and reverse primers. Then add Forward Inner Primer and Backward Inner Primer, with *Bst* DNA polymerase. The tubes containing reaction mixture were incubated at 60 °C for 60 min in water bath. After the incubation any of the visualization method could be employed to check for positive reaction (**Fig. 8**). The LAMP Assay could be performed without DNA extraction by elimination of DNA purification stage and directly using crude leaf samples supernatant as a template (**Fig. 8**).

## 4. INSERTIONAL MUTAGENESIS

Insertional mutagenesis is an alternative means to study gene function by disrupting the gene structure. This method is based on the insertion of foreign DNA into the gene of interest. To investigate a difference in phenotype and correlate with a particular gene, plant should be homozygous (homozygous for the insertion- both the copies of target gene are disrupted). This foreign DNA not only disrupts the expression of the gene into which it is inserted but also acts as a marker for subsequent identification of the mutation. As the T_1_ lines are always heterozygous for the insertion, progeny of T_1_ population (T_2_ plants) shows three possible combinations in the genotypic ratio of 1:2:1. In this only 25% plants are homozygous which contain foreign DNA at both alleles of the target gene.

*Procedure*. Set up the PCR of different samples of DNA using two sets of primers (1st set and 2nd set), after the PCR run the agarose gel and their pattern will clearly depict the zygosity status of particular plant (**Fig. 9**).

*Primer design.* The first set of primers contains two target gene specific primers [F+R]. Forward primer binds to the target gene and reverse primer to regions at the known insertion site (shown in red). The second set of primers contains the same forward primer [F] as first set but reverse primer [R’] are specific to foreign (t-DNA) DNA region.

*Data analysis*:

(1) Wild type: Only primer set 1 will work and hybridize to the specific position as shown in **Figure 9** with a theoretical amplicon size of 300 bp. Whereas the other set of primers will not work because wild type plant lacks insertional t-DNA.

(2) Heterozygous type: Both sets of the primers will work in this case, primer set 1 will hybridize to non-disrupted copy, whereas the reverse primer in set 2 will hybridize to t-DNA region.

(3). Homozygous type: In this case both copies of the genes are disrupted with insertion of t-DNA at specific site. So there only second set of primers will work and give an amplicon (500 bp) larger in size than wild type. Whereas reverse primer in primer set 1 will not work due to disruption of hybridization site (shown in red) by t-DNA insertion.

## DISCUSSION

Genetic engineering is a very powerful and useful tool to create additional genetic diversity that can be further incorporated into crop breeding programs [49]. In general, transgenic events cannot be directly used for cultivation because it shows segregation in the descendent generation. But careful choice of starting material for genetic transformation coupled with precise integration into cultivated genotypes can allow us to reap its full benefits in crop improvement. Transformation of microspore would allow direct production of homozygous transgenic plants [50]. But microspore culture is not easily feasible for most of the crops [51]. To overcome these limitations, researchers have moved toward other transformation methods like in-planta transformation, floral dip and callus regeneration methods. All these transformation methods produce hemizygous condition in T_0_ plant [52], which require special techniques to screen transformants that are homozygous and have single copy insertion. Model plants such as *A. thaliana* can be easily screened with antibiotic marker selection because of its short generation times. This short life span allows the selection of homozygous plants within respectable timelines through Mendelian segregation studies over few generations [53]. However, this marker selection approach is very slow and cumbersome for agricultural models such as wheat, barley, rice and corn. Hence, in these crops, apart from testing for stable incorporation of transgene, there is requirement of methods to govern the zygosity and copy number of transgene. Southern blot analysis offers the testing of transgene insertion, copy number of transgene and the zygosity of transgene simultaneously. All these conventional techniques have some limitations such as labor intensive, expensive, and difficult to scale up for large numbers of samples.

Emergence of competitive PCR and real-time PCR appeared as substitution of conventional methods for zygosity testing. These methods allow rapid detection of homozygosity because they directly depend upon PCR of the amplification of transgene sequences [33]. Competitive PCR relies on final amplification product whereas real-time PCR uses the threshold value of amplicon. Real-time PCR technique requires normalization of the DNA sample with an endogenous reference gene [33, 54] or a standard curve [38, 55]. Due to the error in selection of reference gene and amplification of by-products, sometimes accuracy of real-time PCR is a matter of controversy [38, 54-56]. Although competitive PCR provides the accuracy and a rapid detection of homozygosity [57], but cloning or site directed mutagenesis of transgene to design the tailored DNA makes it a cumbersome technique. Another major drawback for both of these techniques is that it remains restricted to transgenic which have only single site insertion because both of these techniques measure the homozygosity quantitatively and are unable to distinguish between copy number and homozygosity of transgene.

Presently next generation techniques include methods such as next generation sequencing (NGS), dPCR and LAMP assay for the determination of homozygosity. NGS is based on the production of large number of short sequence reads which can be assembled into larger contigs to sequence entire genome. To determine the zygosity, NGS has been adopted for targeted sequencing, in which a particular region is sequenced to determine zygosity status of transgene [45]. To apply targeted sequencing, adjacent genome sequence of transgene must be available. However, for most of the crop, whole genome sequence information is not available. So dPCR is advantageous over targeted sequencing since it is not limited by sequence information [58]. The major advantage of these approaches is that there is no requirement of standard curve [59, 60]. Data generated through these techniques depends on sequence specificity, which makes this technique more reliable than real time PCR and Southern blot analysis. Recent comparative study of dPCR with other techniques like Southern hybridization and real-time PCR by Glowacka *et al*. in 2016 [58, 61] suggested the dPCR as one of the best techniques for zygosity testing in short time duration with a very high accuracy and consistent results. NGS techniques allow easy handling of large number of samples [62] due to the one-time optimization and normalization [28]. These techniques are robust but require highly purified DNA, as slight impurities in the target DNA can affect the results.

LAMP assay, another next generation technique, does not require purified DNA and overcomes the limitation of dPCR and NGS. This technique can be directly applicable on crude plant extract. Unlike Southern hybridization, it requires less DNA and does not involve difficult cloning steps as in competitive PCR. LAMP is a modern technique but still requires typical nested PCR primer combination and UV spectrophotometer for analysis of final product. Although, the result is again based on the final amplified product, which raises a doubt on the accuracy and consistency of results as compared to other two next generation techniques (NGS and dPCR).

Insertional mutagenesis is very common in plants such as *A. thaliana* and rice whose complete genome sequence is available. Determination of homozygosity of insertional mutants is comparatively easy and reliable as compare to the transgene insertion events study. In this case determination of homozygosity is done by only PCR based method. This approach uses different specific primer combinations for amplification and size of amplicon conclude the zygosity status of plants.

To summarize the complete review of all the available techniques for the zygosity testing to acquire homozygosity, it has been suggested that dPCR emerges as most promising technique which provides more accurate, reliable and consistent results comparable to the Southern hybridization in short time duration without any cumbersome procedure and genome sequence limitation. dPCR is a new technique with a lot of scope of improvement in future and may provide more easy and highly reliable results for homozygosity.

While making transformants, single insertion event and homozygosity of transgene are highly desirable. Single insertion lines are also important because sometimes multiple insertion cause silencing of transgene [63]. Single insertion line can be identified in T_0_ generation, whereas homozygous can be achieved at T_1_ generation. With the frequent introduction of new transgenic traits into diverse crops, complementary efforts on breeding through biotechnology are essential, with equivalent funds diversion, in order to not lose the overall progress. Until these approaches are further refined and become universally applicable, the factors associated with the process of tissue culture and transformation will continue to confound the results. Therefore, the approaches outlined above (**Fig. 10**) will remain relevant for meaningful analysis of transgenic plants.

**Table 1.** Summary of the pros and cons of all techniques reviewed in this paper.

Serial No.	Methods	Pros	Cons
1	Antibiotic selection	• Easy and does not require typical instruments (*e.g.*, PCR and gel electrophoresis)	• Difficult to scale up the procedure. • Works in case of single insertion of transgene only. • Transgene should obey the Mendel law of segregation
2	Southern blot hybridization	• Sensitive • Determine both copy number and homozygosity simultaneously	• Requires large amount of DNA sample. Applicable on single and double insertion of transgene.
3	Competitive PCR	• Simple and Reliable • Requires less amount of DNA. • Can easily scale up for large number of samples.	• Requires difficult cloning or site directed mutagenesis
4	Real time PCR	• Requires less amount of DNA • Easy to operate • Can easily scale up • Requires less time for assay	• Impurities in DNA and non-specific products can sometime cause controversial result. • Not applicable when transgene insertion occurs at more than one place.
5	TAIL PCR	• Requires less amount of DNA • Highly specific • Can work even transgene integration occurs more than two sites • Can easily scale up	• Time consuming • Expensive in terms of primer design • Difficult to optimize PCR condition to get specific product.
6	Digital PCR	• More specific than real time PCR • Requires less amount of DNA • Can easily scale up • Requires very less time for assay	• Not applicable, when transgene insertion occurs at more than one place. • Highly expansive • Requires highly purified DNA
7	NGS	• Highly specific • Requires less amount of DNA • Can easily scale up	• Sequence of crop must be known. • Highly expansive • Requires highly purified DNA
8	LAMP	• Can work directly on plant or crude DNA • Can easily scale up	• Requires specific Taq for amplification • Designing nested primer combination can be tedious

## CONCLUSION

Knowledge of zygosity status for a transgene locus is highly essential in plants, especially for those which have longer reproductive cycles. Identification of stable transformants and homozygous lines in initial generations itself is of great importance. Presently, many efficient, iterative and high throughput techniques are available but still most of the laboratories rely on Southern hybridization because it is still the most robust, unambiguous and extensively used method for zygosity testing in plants. However, it is labor intensive and time consuming therefore, this is the right time to shift our gears to the modern next generation techniques like NGS and dPCR for high-throughput selection of transgenic plants. dPCR is emerging as the most appropriate method for zygosity testing due to the reliable and highly consistent results, similar to Southern hybridization, but with advantages such as high speed which puts it ahead of all other available techniques. Thus, based on the review of all the available methods (**Table 1**), we conclude that, dPCR is the most accurate, reliable, precise and fast method in the determination of transgene zygosity. There is a need for acceptance of these techniques which could speed up the screening and characterization of transgenic events. These next generation techniques could be proven to be useful tool to test the hypotheses put forward by basic researchers, and as a valuable tool for applied transgenic research to address the key issues of food security and global environmental changes.
